# Differences in Anatomic Adaptation and Injury Patterns Related to Valgus Extension Overload in Overhead Throwing Athletes

**DOI:** 10.3390/diagnostics14020217

**Published:** 2024-01-19

**Authors:** Kathryn J. Stevens, Akshay S. Chaudhari, Karin J. Kuhn

**Affiliations:** 1 Department of Radiology, Stanford University Medical Center, Palo Alto, CA 94304, USA; akshaysc@stanford.edu; 2 MAPMG: Mid-Atlantic Permanente Medical Group, Rockville, MD 20852, USA; karinj12@gmail.com

**Keywords:** throwing athlete, baseball, valgus extension overload, valgus instability, ulnar collateral ligament, lateral compression forces, subchondral sclerosis, posteromedial shear

## Abstract

The purpose of our study was to determine differences in adaptative and injury patterns in the elbow related to valgus extension overload (VEO) in overhead throwing athletes by age. A total of 86 overhead throwing athletes and 23 controls underwent MRI or MR arthrography (MRA) of the elbow. Throwing athletes were divided by age into three groups: ≤16 years (26 subjects), 17–19 years (25 subjects), and ≥20 years (35 subjects). Consensus interpretation of each MRI was performed, with measurements of ulnar collateral ligament (UCL) thickness and subchondral sclerosis at the radial head, humeral trochlea, and olecranon process. A higher frequency of apophyseal and stress injuries was seen in adolescent athletes and increased incidence of soft tissue injuries was observed in older athletes. Early adaptive and degenerative changes were observed with high frequency independent of age. Significant differences were observed between athletes and controls for UCL thickness (*p* < 0.001) and subchondral sclerosis at the radial head (*p* < 0.001), humeral trochlea (*p* < 0.001), and olecranon process (*p* < 0.001). Significant differences based on athlete age were observed for UCL thickness (*p* < 0.001) and subchondral sclerosis at the olecranon process (*p* = 0.002). Our study highlights differences in anatomic adaptations related to VEO at the elbow between overhead throwing athletes and control subjects, as well as across age in throwing athletes.

## 1. Introduction

Valgus extension overload (VEO) syndrome encompasses a variety of symptoms and clinical findings that occur in predictable patterns related to biomechanical changes occurring in the elbows of overhead throwing athletes [1,2,3,4,5]. Excessive forces are placed on the elbow during the throwing motion, resulting in tensile stresses along the medial elbow, compression forces on the lateral elbow, and shear forces on the posteromedial elbow [6,7]. During the late cocking phase of throwing, the shoulder is placed in maximal external rotation with the elbow flexed. As the throw transitions from the late cocking to early acceleration phases, there is a large transfer of potential energy to kinetic energy as the humerus begins to internally rotate about the shoulder and the elbow extends, generating a large valgus torque across the elbow [1,8]. The anterior bundle of the ulnar collateral ligament provides the main restraint against valgus stress at the elbow, making it particularly susceptible to injury in throwing athletes. A variety of anatomic adaptations can occur in response to repetitive valgus loading, including thickening of the ulnar collateral ligament (UCL) and osseous remodeling [9,10,11,12,13,14]. A spectrum of injuries can occur when these adaptive mechanisms fail as a consequence of compromised medial ligamentous integrity with resultant valgus instability resulting in accentuated loading of the radiocapitellar joint and excessive shear forces on the posteromedial elbow [6].

Elbow injuries occurring in the setting of VEO demonstrate predictable patterns in terms of clinical presentation and imaging findings [15,16]. Due to underlying anatomic differences in skeletally immature versus mature throwing athletes, these patterns manifest differently. Pediatric athletes have open growth plates that are softer and weaker than the ligaments and tendons inserting into them, and therefore demonstrate a propensity for apophyseal, osteochondral, and stress injuries [17,18,19], whereas adult athletes with closed growth plates are at greater risk for soft tissue injury and progressive degenerative change [15,20,21]. 

The purpose of this work was to qualitatively and quantitatively evaluate the anatomic adaptations and spectrum of injury patterns that occur in overhead throwing athletes versus non-throwing control subjects, as well as identify differences in athletes based on age.

## 2. Materials and Methods

This retrospective study was performed in compliance with the Health Insurance Portability and Accountability Act. The requirement for informed consent was waived by our institutional review board in view of the retrospective nature of this study.

### 2.1. Patient Selection

An electronic search engine operating on the institutional radiology information system was used to retrospectively identify all patients who had either a conventional elbow MRI or MR arthrogram (MRA) performed between January 2001 and March 2020. The corresponding medical record of each patient identified was reviewed to determine participation in an overhead throwing sport. A total of 86 athletes were identified that met these criteria for whom age, gender, sport and position, hand dominance, and presenting symptoms were also recorded. A total of 23 age-matched control subjects who did not currently participate in an overhead throwing sport were identified using the same search for whom age, gender, and any sport participation were recorded. 

### 2.2. Retrospective Review of MRI/MRA Images

MRI/MRA examinations were interpreted by an experienced musculoskeletal radiologist with over 20 years of experience and a musculoskeletal radiology fellow. A consensus interpretation was rendered for each subject regarding the presence or absence of specific anatomic adaptations and injury patterns related to medial tension overload, lateral compressive forces, and posteromedial shear forces in the setting of VEO that have been previously described in the literature [15,16] (Table 1).

Fluid in the posteromedial humeroulnar recess has been associated with inflammatory and/or degenerative changes occurring secondary to posteromedial shear forces in the setting of VEO [22] and was defined as the presence of fluid within the joint recess, producing a convex posteromedial margin, with or without synovitis. Scarring in the olecranon fat pad has been demonstrated in up to one third of overhead throwing athletes [23], defined as low signal intensity bands traversing the fat pad. Ulnar neuropathy was defined as an increase in caliber and intrinsic signal intensity of the nerve on three-plane assessment, which included a coronal T2 sequence to avoid false positives related to magic angle phenomenon where the ulnar nerve curves into the cubital tunnel. The remainder of the entities listed in Table 1 were assessed based on standard radiology definitions. 

**Figure 1:**
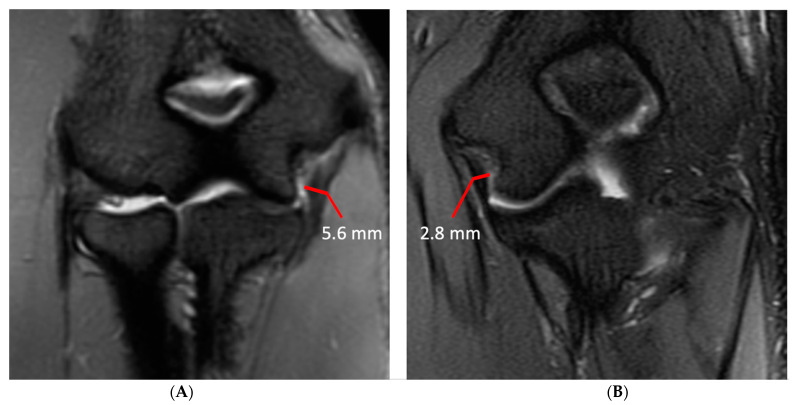
Ulnar collateral ligament measurement technique in two subjects. (**A**) Coronal STIR image of the right elbow in a right-hand-dominant 21-year-old male professional baseball player demonstrating an ulnar collateral ligament with a maximal transverse width of 5.6 mm. (**B**) Coronal T2 fat-saturated image of the left elbow of a 21-year-old left-hand-dominant male non-throwing control demonstrating an ulnar collateral ligament with a maximal transverse width of 2.8 mm.

**Figure 2:**
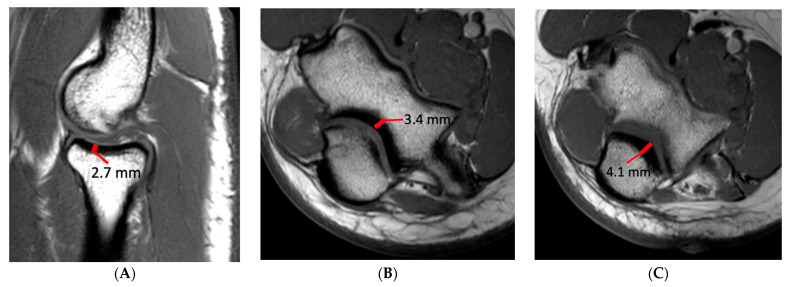
A 16-year-old male baseball pitcher with images demonstrating the measurement technique for quantifying subchondral sclerosis (thick red line). (**A**) Sagittal T1-weighted image demonstrating the measurement technique at the radial head, taken parallel to the articular surface; (**B**) axial T1-weighted image demonstrating the measurement technique at the posterior humeral trochlea, again made parallel to the articular surface; (**C**) axial T1-weighted image demonstrating the measurement technique at the olecranon process.

Injury of the UCL was scored as grade 1, 2 or 3. Injury was considered grade 1 if the ligament fibers were intact but there was periligamentous edema. If there was partial tearing of ligament fibers or abnormal signal within the UCL it was considered grade 2, and if fibers were completely disrupted it was considered grade 3. Ulnar collateral ligament (UCL) thickness and the maximal thickness of subchondral sclerosis in the central radial head, olecranon process, and posterior humeral trochlea were measured independently on separate workstations. UCL thickness was measured at the midpoint of the ligament on coronal fat-saturated T2-weighted or short-tau inversion recovery (STIR) images with a transverse measurement spanning the low signal ligament fibers and excluding any adjacent fat and fluid signal (Figure 1). The thickness of subchondral sclerosis in the radial head was measured on nonfat-saturated sagittal T1-weighted images, making the measurement perpendicular to the articular surface (Figure 2). On MR arthrograms the thickness of subchondral sclerosis in the radial head was measured on coronal non-fat-saturated T1-weighted images, as the only sagittal images acquired were fat-saturated. Subchondral sclerosis along the posterior humeral trochlea and olecranon process was measured on non-fat-saturated axial T1-weighted or proton-density-weighted images at the greatest anteroposterior length (Figure 2). No axial non-fat-saturated images were available in the MR arthrogram studies, but with appropriate windowing the subchondral sclerosis could still be readily identified on the fat-saturated images and was measured on the axial fat-saturated proton-density-weighted images.

### 2.3. Statistical Analysis

Variations in UCL thickness and thickness of subchondral sclerosis in the radial head, olecranon process, and posterior humeral trochlea between overhead throwing athletes and controls were measured using a Mann–Whitney U-Test. Violin plots summarizing the boxplot measurements and distribution of these values were computed. All athletes were divided into three separate subgroups based on age, with roughly equal bin sizes.

Subgroup 1 consisted of individuals aged 16 years or less, subgroup 2 consisted of individuals aged between 17 and 19 years, and subgroup 3 consisted of individuals aged 20 years or older. Variations between UCL thickness and thickness of subchondral sclerosis in the radial head, olecranon process, and humeral trochlea amongst the three groups of athletes were evaluated using Kruskal–Wallis tests with Dunn post hoc tests, if required. 

Intra-reader variation for the measurement of the UCL thickness and the measures of subchondral sclerosis in the radial head, humeral trochlea, and olecranon process was assessed using concordance correlation coefficients and tested using Mann–Whitney U-Tests. Bland–Altman plots were also used to visualize the intra-reader variation and to compute the bias and 95% limits of agreement between the two readers. All statistical analysis was performed in Python (v3.6.1) using the NumPy (v1.12.1) and SciPy (v0.19.1) libraries. Significance levels were set to *p* < 0.05.

## 3. Results

Eighty-six patients met the criteria for inclusion in the overhead throwing athlete group (78 males and 7 females; mean age 18.6 years; range 12–33 years; median, 19 years). Of the 86 athletes, 74 were baseball players (41 pitchers) and 12 played an alternative overhead sport, including water polo (4), javelin (3), softball (2), volleyball (1), tennis (1), football (1). Imaging was performed on the dominant elbow in 84 patients: of these, 7 were professional baseball pitchers, 34 collegiate, and 45 amateur or little-league athletes. The most common symptom was medial elbow pain, seen in 57 (66%) of athletes, with chronic pain in 43 (75%) and acute pain in 14 (25%).

Subgroup analysis stratifying athletes by age yielded 26 subjects in subgroup 1 (25 males and 1 female; mean age 14.9 years; range 12–16 years), 25 subjects in subgroup 2 (24 males and 1 female; mean age 18.4 years; range 17–19 years), and 35 subjects in subgroup 3 (30 males and 5 females; mean age 21.4 years; range 20–33 years).

Twenty-three subjects were included in the control group (17 males and 6 females; mean age 20.8 years; range 13–28 years), none of whom had a history of regular participation in an overhead throwing sport recorded in their electronic medical record.

The results of the consensus qualitative assessment of specific anatomic adaptations and injury patterns in the overhead athlete group stratified by age and in aggregate are presented in Table 2. 

### 3.1. Qualitative Changes

**Figure 3:**
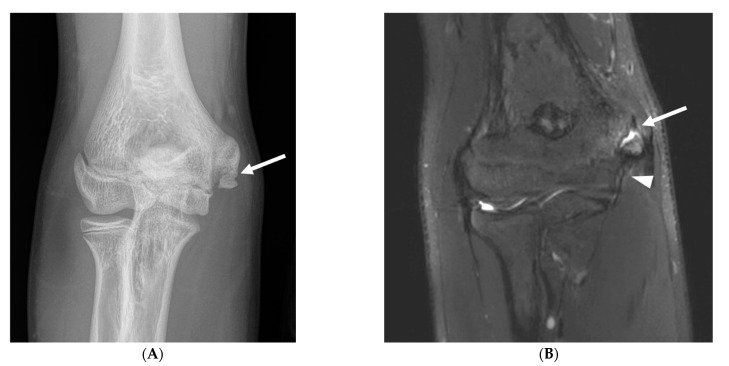
A 12-year-old right-hand-dominant male baseball pitcher who developed acute medial elbow pain during a pitching lesson. (**A**) AP radiograph demonstrates an acute avulsion fracture of the medial epicondylar apophysis (arrow). (**B**) Coronal fat-saturated T2-weighted image demonstrates an acute avulsion fracture of the medial epicondylar apophysis (arrow). The anterior band of the ulnar collateral ligament remains intact (arrowhead).

**Figure 4:**
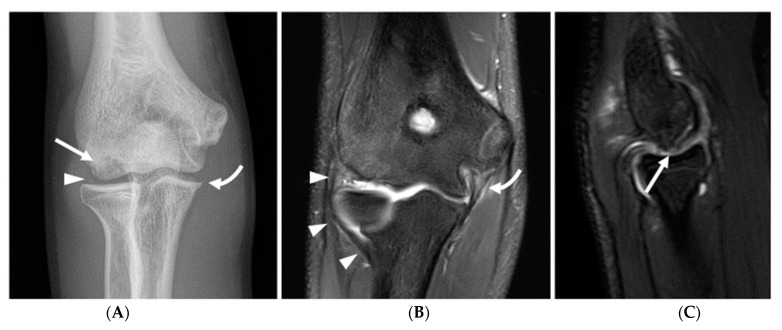
A 16-year-old male right-hand-dominant baseball pitcher presenting with chronic right lateral elbow pain and acute medial pain after pitching. (**A**) AP radiograph demonstrating irregular lucency in the capitellum (arrow), compatible with osteochondritis dissecans (OCD), and a punctate joint body is seen in the radiocapitellar joint space (arrowhead). A small ulnar traction spur is visible along the sublime tubercle (curved arrow). (**B**) Coronal fat-saturated proton-density-weighted image demonstrates thickening and of the ulnar collateral ligament (curved arrow), with intrinsic high T2 signal and periligamentous edema, compatible with grade 2 injury. Of note, the lateral ulnar collateral ligament is intact and of normal morphology (arrowheads). (**C**) Sagittal fat-saturated T2 Cube image demonstrates an osteochondral lesion along the anterior capitellum with disruption of the articular cartilage and subchondral bone plate (arrow), and high T2 signal deep to the subchondral bone plate, indicating an unstable lesion.

Osseous abnormalities resulting from valgus extension overload were seen more frequently in younger athletes. Medial epicondylar apophysitis and acute apophyseal avulsion injuries from medial tension overload were only observed in athletes in subgroup 1 (Figure 3), although evidence of chronic avulsion injury was seen in the older age groups. Osseous findings from radiocapitellar overload were also seen most frequently in athletes in subgroup 1 (Figure 4), whereas posterior humeroulnar chondral injuries occurred with greatest frequency in subgroup 2.

**Figure 5:**
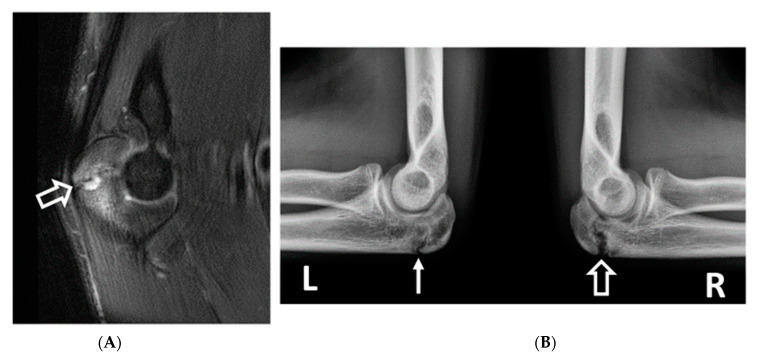
A 15-year-old right-hand-dominant male baseball pitcher with right posterior elbow pain. (**A**) Sagittal fat-saturated T2-weighted image of the right elbow demonstrates irregularity, increased T2 signal and cystic change along the olecranon physis (open arrow), with periphyseal bone marrow edema, compatible with olecranon apophysitis. (**B**) Radiographic comparison of the bilateral elbows demonstrates physeal widening, irregularity, and delayed closure of the physis (open arrow) compared with the physis of the asymptomatic left elbow (arrow), which is partially fused superiorly.

**Figure 6:**
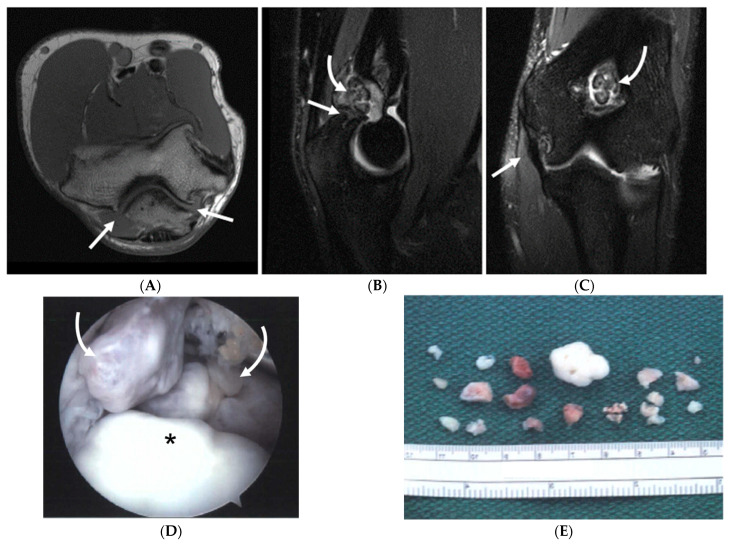
A 19-year-old male collegiate right-hand-dominant baseball pitcher with mechanical symptoms in the right elbow for 2–3 years and an inability to extend elbow. (**A**) Axial T1-weighted image demonstrates joint space narrowing in the posterior humeroulnar joint, with prominent subchondral sclerosis and small marginal osteophytes (arrows). (**B**) Sagittal fat-saturated T2-weighted image demonstrates multiple joint bodies in the olecranon fossa (curved arrow) and fragmentation of the olecranon tip (arrow). (**C**) Coronal fat-saturated T2-weighted image demonstrates marked thickening of the anterior band of the ulnar collateral ligament (arrow). Joint bodies are again seen in the olecranon fossa (curved arrow). (**D**) Arthroscopic image demonstrating hypertrophy of the olecranon process (asterisk) and multiple joint bodies (curved arrow). (**E**) Photograph showing the multiple joint bodies retrieved at surgery.

Olecranon apophysitis was also only seen in athletes in subgroup 1 (Figure 5), with the exception of one athlete in subgroup 3 who had a chronic injury with associated nonunion of the olecranon apophysis, and olecranon stress reaction or fractures were observed with a higher frequency in subgroups 1 and 2 (12% and 24%, respectively), with a chronic injury seen in a single subject in subgroup 3. Other findings consistent with repetitive loading secondary to posteromedial shear and resultant degenerative changes in the posterior humeroulnar joint were observed with high frequency across all age groups, although this progressively increased with age. Specifically, humeroulnar osteophytes were observed in 62% of subjects in subgroup 1, 72% of subjects in subgroup 2, and 74% of subjects in subgroup 3 (Figure 6). This is in contrast to controls, where only 17% of subjects demonstrated posterior humeroulnar osteophytes. 

Although grade 1 UCL injuries occurred with similar frequency across age groups, grade 3 UCL injuries were only seen in athletes in subgroup 3. Similarly, grade 2 UCL injuries occurred with greater frequency in older athletes (48% of subjects in subgroup 2 and 49% in subgroup 3, compared to 23% in subgroup 1). No evidence of UCL injury was observed in control subjects. Flexor–pronator, common extensor, triceps, and biceps/brachialis tendinopathy and injury demonstrated a similar pattern with the greatest frequency observed in athletes in subgroup 3.

In contrast, these findings were relatively uncommon in the control group with ulnar traction spurs seen in six subjects (26%) and ulnar neuropathy seen in four subjects (17%). The four control subjects with imaging findings consistent with ulnar neuropathy were all clinically symptomatic, which was in contrast to the relatively small percentage of clinically symptomatic overhead athletes (9 out of 32 or 28%). 

A comparison of the most frequently encountered imaging findings in athletes versus control subjects is presented in Table 3.

Although grade 1 UCL injuries occurred with similar frequency across age groups, grade 3 UCL injuries were only seen in athletes in subgroup 3. Similarly, grade 2 UCL injuries occurred with greater frequency in older athletes (48% of subjects in subgroup 2 and 49% in subgroup 3, compared to 23% in subgroup 1). No evidence of UCL injury was observed in control subjects. Flexor-pronator–pronator, common extensor, triceps, and biceps/brachialis tendinopathy and injury demonstrated a similar pattern with the greatest frequency observed in athletes in subgroup 3.

**Figure 7:**
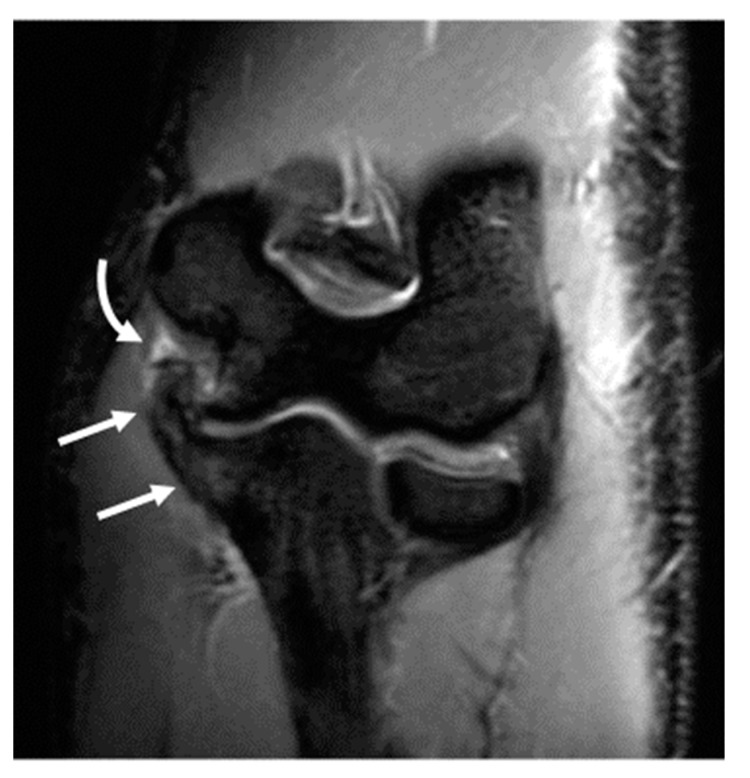
A 20-year-old male left-hand-dominant Major League Baseball pitcher. Coronal STIR image of the left elbow demonstrates a large ulnar traction spur with associated bone marrow edema (arrow). There is full thickness tear of the posterior band of the ulnar collateral ligament (curved arrow).

Other findings expected to occur with repetitive stresses over time, specifically ulnar neuropathy and development of an ulnar traction spur at the UCL insertion into the sublime tubercle, demonstrated an expected trend of increasing frequency with age. Ulnar traction spurs were pronounced in several athletes in subgroup 3 with professional experience (Figure 7). 

### 3.2. Quantitative Changes

**Figure 8:**
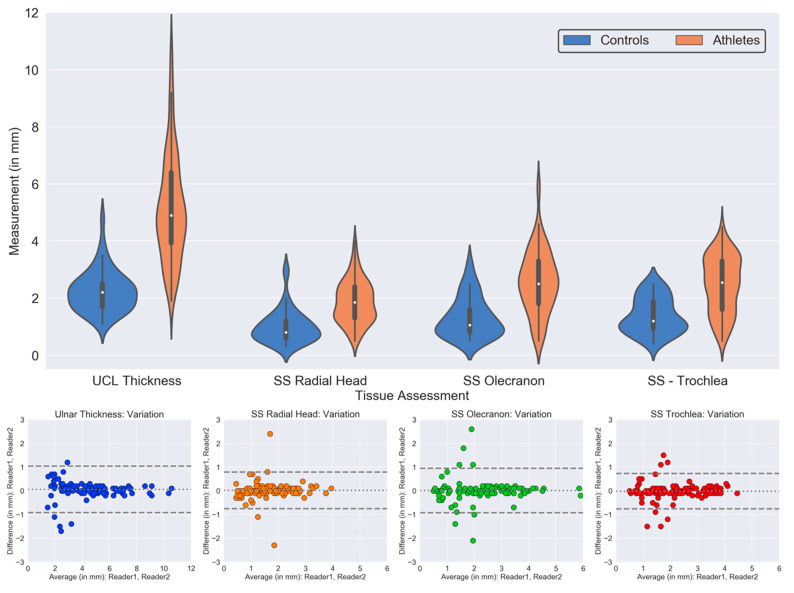
Graphic comparison of quantitative differences in UCL thickness and subchondral sclerosis (SS) at the radial head, posterior humeral trochlea, and olecranon process between case and control subjects, with Bland–Altman visualization for intra-reader variation.

The results of quantitative analysis comparing group differences in UCL thickness and subchondral sclerosis thickness in the radial head, posterior humeral trochlea, and olecranon process between overhead athletes and control subjects are presented in Table 4 and graphically depicted in Figure 8.

As seen in Table 4, a significant difference between groups exists for mean UCL thickness, with overhead athletes demonstrating a mean UCL thickness and standard deviation of 5.1 ± 1.8 mm and control subjects demonstrating a mean UCL thickness of 2.3 ± 0.8 mm (*p* = 0.002). When controlling for differences between groups based on degree of UCL injury, there was also a significant difference between groups, with overhead athletes presenting with a grade 1 UCL injury, i.e., mild periligamentous edema but no abnormal signal within the ulnar nerve demonstrating a mean UCL thickness of 4.4 ± 1.4 mm and control subjects demonstrating a mean UCL thickness of 2.3 ± 0.8 mm (*p* < 0.001). Similarly, there was a significant difference between groups when excluding grade 3 UCL injuries, with overhead athletes demonstrating a mean thickness of 5.1 ± 1.8 mm and control subjects demonstrating a mean thickness of 2.3 ± 0.8 mm (*p* < 0.001).

Comparison of group differences between athletes across age for UCL thickness and subchondral sclerosis at the radial head, posterior humeral trochlea, and olecranon process are presented in Table 5. There were no significant differences between any of the three subgroups for thickness of subchondral sclerosis in the radial head (*p* = 0.09) or posterior humeral trochlea (*p* = 0.27). However, significant differences were found for UCL thickness between subgroups 1 and 2 (*p* = 0.01) along with subgroups 1 and 3 (*p* < 0.001), but not between subgroups 2 and 3 (*p* = 0.14). Similarly, significant differences were found for thickness of subchondral sclerosis in the olecranon process between subgroups 1 and 2 (*p* = 0.03) along with subgroups 1 and 3 (*p* < 0.001), but not between subgroups 2 and 3 (*p* = 0.40).

There was high concordance (greater than 90%) between measurements on inter-rater reliability analysis (Pearson coefficients > 0.99, lowest *p*-values = 0.79) across measurements of UCL thickness and subchondral sclerosis along the radial head, humeral trochlea, and olecranon process, as well as intra-rater reliability analysis (Table 6). 

## 4. Discussion

Valgus extension overload (VEO) in the throwing athlete contributes to repetitive loading of soft tissue and osseous structures at the elbow that results in predictable clinical and imaging patterns. Early biomechanical studies established that the UCL functions as the primary stabilizer at the elbow during the majority of the throwing motion [8,24]. In the setting of repetitive microtrauma, progressive UCL injury, and resultant valgus instability, the biomechanical distribution of forces at the elbow is altered [25,26], resulting in predictable injury patterns at the elbow that can be readily diagnosed at imaging [15,16]. This alteration in force distribution also likely contributes to the soft tissue and osseous adaptations seen at the elbow, as well as early and progressive degenerative changes with continued play. A number of authors have previously reported on specific anatomic adaptations that occur secondary to the application of repetitive stresses at the elbow. Atanda et al. [9] demonstrated a progressive increase in mean UCL thickness on stress ultrasound with age and number of years of professional participation in 127 asymptomatic professional baseball pitchers aged 17–21 years. Tanaka et al. [12] compared physical and clinical examination results with corresponding MR imaging in 64 asymptomatic adolescent baseball players aged 9–13 years, revealing asymptomatic adaptive changes in the UCL in 53.1% of subjects. Kooima et al. [21] prospectively reviewed MRI examinations of the dominant and nondominant elbow in 16 asymptomatic professional baseball players, with 81% demonstrating asymmetric thickening or abnormal signal in the UCL and 81% showing evidence of posteromedial shear with subchondral sclerosis and spurring in the dominant versus non-dominant elbow. Hurd et al. [27] performed MRI scans of the elbow in 23 asymptomatic high-school baseball pitchers with a mean age of 16 years, and 65% demonstrated thickening of the anterior band of the ulnar collateral ligament and 61% showed subchondral sclerosis of the posteromedial trochlea. 

Similarly, other authors have assessed adaptive changes in the osseous structures of the elbow. Yoshizawa et al. [13] demonstrated asymmetric enlargement of the medial epicondylar ossification center in the dominant compared to the nondominant elbow in 80 adolescent baseball players aged 9–14 years, suggesting that the UCL may induce excessive repetitive tensile stress on the medial epicondyle. Sada et al. [14] showed an asymmetric increase in humerocapitellar volumetric bone mineral density and trabecular thickness in the dominant versus nondominant elbow of 17 baseball players aged 16–29 years, likely the result of repetitive valgus stresses generated by pitching. 

Our study showed similar specific adaptive changes in soft tissue and osseous structures with significant differences between overhead athletes and control subjects, but to the authors’ knowledge, this is the first study to qualitatively and quantitatively examine differences by age across adolescent to adult overhead throwing athletes compared to controls by MR imaging. The results of the qualitative analysis of differences in injury patterns in adolescent to adult players stratified by age have been presented in Table 2 and are in keeping with prior studies that demonstrate unique injury patterns in adolescent players, with an increased risk for apophyseal and stress injuries [17,19]. In general, osseous abnormalities, including osteochondritis dissecans of the capitellum and radiocapitellar osteoarthrosis with joint bodies, were observed with greater frequency in younger athletes, with the exception of chondral injuries and bony reactive changes in the posteromedial humeroulnar joint, which were seen with high frequency across all age groups, but trended higher in the older overhead athletes. 

Soft tissue abnormalities including ligamentous and tendon pathology were observed with greater frequency in the older subgroups. Although low grade UCL injuries were seen in throwing athletes across all age groups, complete UCL tears were only seen in the older athletes in subgroup 3. In a study by Garcia et al. [28] on asymptomatic professional baseball pitchers, signal alterations of the UCL on MRI were associated with detrimental effects on pitching throughout the remainder of the player’s career in Major League baseball. Signal alterations in the UCL and other asymptomatic MRI abnormalities, including small partial tears of the proximal UCL and findings associated with posteromedial impingement also helped to predict future injury status and need for future elbow surgery. 

Ulnar nerve pathology can occur in throwing athletes secondary to traction from valgus stress, compression from scar tissue, posteromedial osteophytes, or flexor muscle hypertrophy, as well as friction due to subluxation of the ulnar nerve [7]. Ulnar nerve abnormalities were seen in 32 of 86 (37%) of our overhead throwing athletes, but only 9 of the 32 athletes had symptoms of ulnar neuropathy. In contrast, ulnar nerve abnormalities were seen in four control subjects (17%), all of whom were clinically symptomatic. This suggests that the changes in the ulnar nerve seen on imaging in overhead athletes as a consequence of VEO may be subclinical in some athletes.

Findings that were relatively infrequent in our study cohort regardless of age were lateral ligamentous injury, heterotopic ossification due to prior UCL injury, osteochondral lesions, osteochondritis dissecans, and observation of a thickened radiocapitellar plica. 

The quantitative analysis of between group differences in UCL thickness and subchondral sclerosis at the radial head, humeral trochlea, and olecranon process demonstrated a significant difference between athlete and control groups across all metrics, supporting the notion that these observed anatomic differences occur secondary to repetitive stresses applied during the throwing motion related to VEO. Similar observations were reported by Kooima et al. [21] in terms of UCL thickness and subchondral sclerosis at the posterior humeroulnar joint. However, no previous studies have reported on subchondral sclerosis at the radial head, and the significant difference between overhead throwing athletes and control subjects suggests an anatomic adaptation reflecting lateral compressive forces at the elbow.

In subgroup analysis of between group differences in athletes across age, UCL thickness and subchondral sclerosis at the olecranon process were observed to increase with age, with a significant difference observed between athletes in subgroup 1 and those in subgroups 2 and 3. No significant difference was observed in UCL thickness or subchondral sclerosis at the olecranon between athletes in subgroups 2 and 3. This observation may indicate that adaptive changes occur early with observed differences becoming less pronounced in older adolescent and young adult athletes, or alternatively, the sample size may have been too small to detect a difference. No significant difference between groups was observed for subchondral sclerosis at the posterior humeral trochlea or radial head, indicating that these findings may be a less sensitive indicator of the biomechanical outcomes of VEO over time.

There were several limitations to our study. The first limitation is the retrospective methodology performed at a single institution using a relatively small sample size. Although clear trends were observed in quantitative analysis between the overhead throwing athlete and control group, a more robust analysis of between group differences across age may have been possible with a larger sample size. In addition, the electronic medical records often did not contain a detailed history of the overhead throwing activities, such as the starting age, years of participation, number of pitches, and level of participation for non-professional and collegiate athletes. A third limitation was the observational methodology used for qualitative analysis of anatomic differences and injury patterns in overhead athletes, which is necessarily subjective. Additionally, as the readers were not blinded to whether subjects were in the athlete or control group, which may introduce a source of bias. A fourth limitation was the small number of age-matched control subjects as well as the greater number of females in the control versus overhead athlete group. However, the availability of control subjects that met our inclusion criteria was limited, as the majority of patients having MRI of the elbow in these age groups were symptomatic throwing athletes with a male preponderance. Although there was no history of overhead throwing activities listed in the medical records in the controls, it may be that some of our controls had participated in overhead throwing sports at a younger age. An additional limitation was inclusion of both MRI and MRA examinations in this study, necessitating some differences in quantitative measurement using an alternative plane or sequence; however, MRA examinations comprised a small proportion of exams evaluated in this study (9 out of a total of 109 studies including both the overhead throwing athletes and control group), with measurements appearing similar across the two imaging modalities. Another limitation was the relative difficulty in comparing UCL thickness in athletes and controls in the setting of concomitant injury. Athletes with grade 3 UCL injury were excluded from analysis in order to decrease the potential for spurious increase in UCL thickness due to retracted ligament fibers. Finally, the overhead athlete group was not completely homogeneous with inclusion of athletes participating in different sports, although it is noted that VEO is known to occur in athletes participating in repetitive overhead activities other than baseball [4,5,29,30].

## 5. Conclusions

The biomechanical stresses that occur at the elbow in throwing athletes secondary to valgus extension overload result in a spectrum of anatomic adaptations and injury patterns that differ based on age. Significant differences are observed with respect to specific adaptations in UCL thickness and subchondral sclerosis at the radial head, posterior humeral trochlea, and olecranon process in throwing athletes compared to control subjects. Differences between groups are also observed in younger versus older athletes in terms of UCL thickness and subchondral sclerosis at the olecranon process, although no significant age-related differences were observed between groups for subchondral sclerosis at the olecranon or radial head. Early adaptive and degenerative changes were observed in athletes independent of age and were progressive with advancing age.

## Figures and Tables

**Table 1:** Specific anatomic adaptations and injury patterns evaluated.

Mechanism	Anatomic Structures and Patterns of Injury
**Medial tension** **overload**	Medial epicondylar apophysitis Medial epicondylar avulsion injury Medial epicondylar bone marrow edema Ulnar collateral ligament thickness Ulnar collateral ligament (anterior bundle) injury and grade Heterotopic ossification related to prior UCL injury Ulnar neuropathy Flexor–pronator tendinopathy or injury Ulnar traction spur at the insertion of the UCL into the sublime tubercle
**Lateral compressive forces**	Panner’s disease (osteochondrosis of the humeral capitellum) Osteochondritis dissecans (fragmentation of the humeral capitellar articular surface) Osteochondral lesion Thickened radiocapitellar plica Subchondral sclerosis along the radial head Radiocapitellar osteoarthrosis and presence of joint bodies
**Posteromedial shear forces**	Olecranon apophysitis Olecranon stress reaction or fracture Subchondral sclerosis along the humeral trochlea and olecranon Humeroulnar chondral injury and/or joint bodies Humeral trochlear or olecranon bone marrow edema Humeroulnar osteophytes Fluid and/or synovitis in the posteromedial humeroulnar recess Scarring in the olecranon fat pad
**Other**	Lateral ligamentous injury Common extensor tendinopathy or injury Triceps tendinopathy or injury Biceps/brachialis tendinopathy or injury

**Table 2:** Comparison of specific anatomic adaptations and injury patterns by qualitative assessment.

Mechanism	Subgroup ≤16 Years (26 Subjects)	Subgroup 17–19 Years (25 Subjects)	Subgroup ≥20 Years (35 Subjects)	Total (86 Subjects)
**Medial tension overload**				
Medial epicondylar apophysitis	6 (23%)	0 (0%)	0 (0%)	6 (7%)
Medial epicondylar avulsion injury	3 (12%)	3 (12%)	1 (3%)	7 (8%)
Medial epicondylar bone marrow edema	8 (31%)	12 (48%)	9 (26%)	29 (34%)
Ulnar collateral ligament injury	17 (65%)	22 (88%)	32 (91%)	71 (83%)
Grade 1 (low grade sprain)	11 (42%)	10 (40%)	11 (31%)	32 (37%)
Grade 2 (partial tear)	6 (23%)	12 (48%)	17 (49%)	35 (41%)
Grade 3 (complete disruption)	0 (0%)	0 (0%)	4 (11%)	4 (5%)
Heterotopic ossification due to prior UCL injury	0 (0%)	1 (4%)	0 (0%)	1 (1%)
Ulnar neuropathy	7 (27%)	10 (40%)	15 (43%)	32 (37%)
Flexor–pronator tendinopathy or injury	11 (42%)	9 (36%)	20 (57%)	40 (47%)
Ulnar traction spur	12 (46%)	15 (60%)	21 (60%)	48 (56%)
**Lateral compressive forces**				
Panner’s disease	0 (0%)	0 (0%)	0 (0%)	0 (0%)
Osteochondritis dissecans	4 (15%)	0 (0%)	0 (0%)	4 (5%)
Osteochondral lesion	1 (4%)	0 (0%)	1 (3%)	2 (2%)
Thickened radiocapitellar plica	2 (8%)	0 (0%)	1 (3%)	3 (3%)
Radiocapitellar osteoarthrosis, joint bodies	5 (19%)	1 (4%)	1 (3%)	7 (8%)
**Posteromedial shear forces**				
Olecranon apophysitis	5 (19%)	0 (0%)	1 (3%)	6 (7%)
Olecranon stress reaction or fracture	3 (12%)	5 (20%)	1 (3%)	9 (10%)
Humeroulnar chondral injury, joint bodies	5 (19%)	12 (48%)	12 (34%)	29 (34%)
Trochlear or olecranon subchondral bone marrow edema/reactive change	9 (35%)	11 (44%)	7 (20%)	27 (31%)
Humeroulnar osteophytes	16 (62%)	18 (72%)	26 (74%)	60 (70%)
Fluid and/or synovitis in the humeroulnar posteromedial recess	6 (23%)	12 (48%)	12 (34%)	30 (35%)
Scarring in the olecranon fat pad	12 (46%)	14 (56%)	12 (34%)	38 (44%)
**Other**				
Lateral ligamentous injury	1 (4%)	1 (4%)	2 (6%)	4 (5%)
Common extensor tendinopathy or injury	4 (15%)	4 (16%)	11 (31%)	19 (22%)
Triceps tendinopathy or injury	3 (12%)	6 (24%)	17 (49%)	26 (30%)
Biceps/brachialis tendinopathy or injury	0 (0%)	1 (4%)	2 (6%)	3 (3%)

**Table 3:** Frequency of imaging findings in athlete and control subjects.

Imaging Finding	Athletes (86 Total)	Control Subjects (23 Total)
Ulnar collateral ligament injury (any grade)	71 (83%)	0 (0%)
Humeroulnar osteophytes	60 (70%)	4 (17%)
Ulnar traction spur	48 (56%)	6 (26%)
Flexor–pronator tendinopathy or injury	40 (47%)	0 (0%)
Scarring in the olecranon fat pad	38 (44%)	2 (9%)
Ulnar neuropathy (total)	32 (37%)	4 (17%)
Symptomatic ulnar neuropathy	9 (10%)	4 (17%)

**Table 4:** Comparison of anatomic adaptations across athlete and control subjects.

Anatomic Adaptation	Athletes	Control Subjects	*p*-Value
Ulnar collateral ligament thickness (mm)	5.1 ± 1.8	2.3 ± 0.8	<0.001
Subchondral sclerosis thickness radial head (mm)	1.9 ± 0.8	1.0 ± 0.6	<0.001
Subchondral sclerosis humeral trochlea (mm)	2.5 ± 1.0	1.3 ± 0.6	<0.001
Subchondral sclerosis olecranon process (mm)	2.6 ± 1.1	1.3 ± 0.7	<0.001

**Table 5:** Comparison of mean anatomic adaptations across athletes stratified by age.

Anatomic Adaptation	Subgroup ≤16 Years	Subgroup 17–19 Years	Subgroup ≥20 Years	*p*-Value **
Ulnar collateral ligament thickness (mm)	4.4 ± 1.9	5.2 ± 1.7 *	5.7 ± 1.7 *	<0.001
Subchondral sclerosis thickness in radial head (mm)	1.8 ± 0.8	1.8 ± 0.7	2.0 ± 0.7	0.09
Subchondral sclerosis in humeral trochlea (mm)	2.2 ± 1.0	2.6 ± 1.1	2.9 ± 1.1	0.27
Subchondral sclerosis in olecranon process (mm)	2.3 ± 1.1	2.6 ± 0.9 *	2.5 ± 0.9 *	0.002

* indicates statistically significant differences compared to subgroup of athletes aged under 16 years via Kruskal–Wallis tests and Dunn’s post hoc assessment; ** *p*-value corresponds to any intergroup difference assessed using Kruskal–Wallis test.

**Table 6:** Comparison of intra-reader variation for the assessment of mean anatomic adaptations.

	Concordance Correlation Coefficient	*p*-Value	Bias	95% Limits of Agreement
Ulnar collateral ligament thickness (mm)	0.97	0.84	0.06	±0.98
Subchondral sclerosis thickness in radial head (mm)	0.88	0.86	0.02	±0.77
Subchondral sclerosis in humeral trochlea (mm)	0.91	0.94	0.01	±0.94
Subchondral sclerosis in olecranon process (mm)	0.93	0.90	−0.02	±0.75

## Data Availability

Data supporting the reported results are with the corresponding author.
